# Epicrania fugax with backward radiation

**DOI:** 10.1007/s10194-011-0407-4

**Published:** 2011-12-21

**Authors:** Juan A. Pareja, Mónica Álvarez, Teresa Montojo

**Affiliations:** Department of Neurology, University Hospital Fundación Alcorcón, Budapest 1, 28922 Alcorcón, Madrid Spain

Dear Editor,

The article by Mulero et al. [1] describing a short series of *epicrania fugax* (EF) with backward radiation added essential knowledge, and we would like to emphasize the diagnostic relevance of EF with backward radiation.

The first 20 reported cases of EF [2, 3] had posterior origins of pain with forwards trajectories up to the ipsilateral eye or nostril. Later, Cuadrado et al. [4] described the first two cases with opposite, backward radiation of the pain. Mulero et al. [1] presented a series of EF stemming from anterior regions of the head with backward trajectories up to the occipital area. This observation not only substantiates the clinical picture of EF but also sharpens its nosologic limits with other similar headaches and neuralgias.

Patients with occipital neuralgia have been considered to have pain spreading similar to EF, but were these cases really occipital neuralgia? Mulero et al. [1] provides us with a clarification as all of the patients had the typical features of EF with pain stemming from anterior regions of the head and spreading backward up to the occipital area, and by no means can this movement be considered occipital neuralgia.

The present and previous observations have outlined EF as a distinctive condition characterized by unilateral paroxysms of head pain felt in motion from onset to end, lasting 1–10 s, starting and ending in territories of different cranial nerves. Forward pain stems from the posterior cranial area, tending to reach the ipsilateral eye or nose. At the end of the attacks, ipsilateral autonomic signs, such as lacrimation, conjunctival injection, or rhinorrhoea, may occur. Backward pain starts in a frontal or periorbital area tending to reach the ipsilateral occipital region. The origin and ending points are constant in each patient. Although attacks are usually spontaneous, they may occasionally be triggered by touching the area where pain originates, which may remain tender between attacks.

EF emerges as a primary headache with a clear-cut clinical picture that can be differentiated clinically from other headaches and neuralgias.
